# pH and Design on *n*–Alkyl Alcohol Bulk Liquid Membranes for Improving Phenol Derivative Transport and Separation

**DOI:** 10.3390/membranes12040365

**Published:** 2022-03-26

**Authors:** Paul Constantin Albu, Szidonia-Katalin Tanczos, Andreea Ferencz (Dinu), Andreia Pîrțac, Alexandra Raluca Grosu, Dumitru Pașcu, Vlad-Alexandru Grosu, Constantin Bungău, Aurelia Cristina Nechifor

**Affiliations:** 1 Radioisotopes and Radiation Metrology Department (DRMR), IFIN Horia Hulubei, 023465 Măgurele, Romania; paulalbu@gmail.com; 2 Department of Bioengineering, University Sapientia of Miercurea-Ciuc, 500104 Miercurea-Ciuc, Romania; tczszidonia@yahoo.com; 3 Analytical Chemistry and Environmental Engineering Department, University Politehnica of Bucharest, 011061 Bucharest, Romania; andra_d24@yahoo.com (A.F.); andreia.pascu@yahoo.ro (A.P.); andra.grosu@upb.ro (A.R.G.); dd.pascu@yahoo.com (D.P.); 4 Department of Electronic Technology and Reliability, Faculty of Electronics, Telecommunications and Information Technology, University Politehnica of Bucharest, 061071 Bucharest, Romania; 5 Department of Engineering and Management, Faculty of Management and Technological Engineering, University of Oradea, 410087 Oradea, Romania; bungau@uoradea.ro

**Keywords:** liquid membrane design, permeation module design, pH operational parameter, bulk liquid membranes, *n*–octanol membranes, *n*–decanol membranes, phenol derivative transports, phenol derivative separation

## Abstract

Regardless of the type of liquid membrane (LM) (Bulk Liquid Membranes (BLM), Supported Liquid Membranes (SLM) or Emulsion Liquid Membranes (ELM)), transport and separation of chemical species are conditioned by the operational (OP) and constructive design parameters (DP) of the permeation module. In the present study, the pH of the aqueous source phase (SP) and receiving phase (RP) of the proposed membrane system were selected as operational parameters. The mode of contacting the phases was chosen as the convective transport generator. The experiments used BLM-type membranes with spheres in free rotation as film contact elements of the aqueous phases with the membrane. The target chemical species were selected in the range of phenol derivatives (PD), 4–nitrophenol (NP), 2,4–dichlorophenol (DCP) and 2,4–dinitrophenol (DNP), all being substances of technical-economic and environmental interest. Due to their acid character, they allow the evaluation of the influence of pH as a determining operational parameter of transport and separation through a membrane consisting of *n*–octanol or *n*–decanol (*n*–AlcM). The comparative study performed for the transport of 4–nitrophenol (NP) showed that the module based on spheres (Ms) was more performant than the one with phase dispersion under the form of droplets (Md). The sphere material influenced the transport of 4–nitrophenol (NP). The transport module with glass spheres (Gl) was superior to the one using copper spheres (Cu), but especially with the one with steel spheres (St). In all the studied cases, the sphere-based module (Ms) had superior transport results compared to the module with droplets (Md). The extraction efficiency (EE) and the transport of 2,4–dichlorophenol (DCP) and 2,4–dinitrophenol (DNP), studied in the module with glass spheres, showed that the two phenolic derivatives could be separated by adjusting the pH of the source phase. At the acidic pH of the source phase (pH = 2), the two derivatives were extracted with good results (EE > 90%), while for pH values ranging from 4 to 6, they could be separated, with DCP having doubled separation efficiency compared to DNP. At a pH of 8 in the source phase, the extraction efficiency halved for both phenolic compounds.

## 1. Introduction

**Figure 1:**
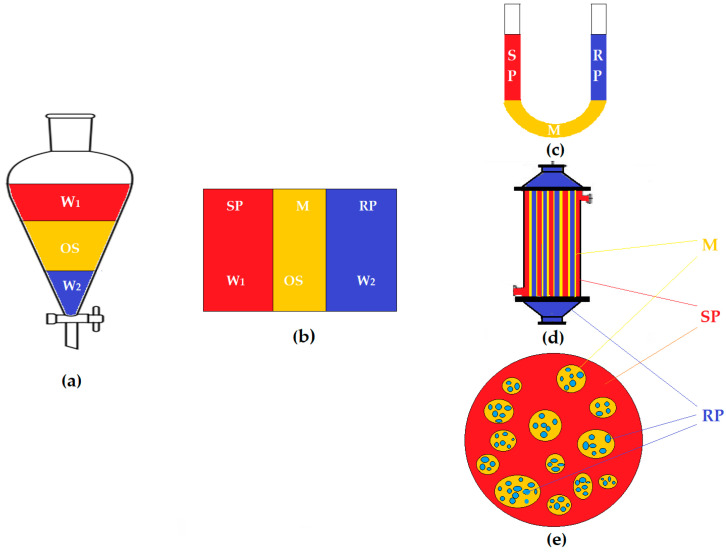
Schematic presentation of extraction and membrane systems with organic solvent: (**a**) water 1 (W_1_), organic solvent (OS), water extraction (W_2_); or (**b**) liquid membranes (LM); (**c**) bulk liquid membranes (BLM); (**d**) supported liquid membranes (SLM); (**e**) emulsion liquid membranes (ELM). Legend: M = membrane; SP = source phase; RP = receiving phase.

Solvent extraction has been a major contribution to the separation, concentration or purification of various chemical species of technical, economical or environmental protection interest [1,2]. The confirmed advantages of extraction with organic solvents have often been limited by the large amount of solvent required for the separation process, mainly due to the two-stage development: extraction and stripping [3,4]. Most attempts to translate the discontinuous process into a continuous one have stopped due to the difficulties of operation and those of making the extractors [5,6]. The concept of transformation of extraction–stripping (Figure 1a), which involves two aqueous phases, has been called the liquid membrane separation process (M), and the apparatus in which this process takes place, the permeation module [7,8]. The two phases involved, source phase (W_1_ or SP) and receiving phase (W_2_ or RP), are separated by the organic solvent (SO) in a continuous unitary process (Figure 1b) in which the chemical species of interest passes from one aqueous phase to the other, selectively, through the organic solvent.

In the extraction with solvents, the aqueous source phase and the organic solvent contact each other intimately through vigorous stirring in a dedicated extractor, after which the organic solvent containing the recovered chemical species is separated (a step that has a relatively long duration). The solvent containing the chemical species of interest is introduced in a stripping apparatus, where it is contacted with a receiving aqueous phase (significantly smaller in volume than the source phase) in order to recover and concentrate the target chemical species [9,10]. However, stripping also takes place over a long period of time, being, in fact, a re-extraction [11]. By operating the two stages of extraction continuously (Figure 1b), the operating time was practically halved, while the amount of organic solvent required for the process was continuously reduced, passing from bulk liquid membranes (BLM) (Figure 1c) to supported liquid membranes (SLM) (Figure 1d), and then to emulsion liquid membranes (ELM) (Figure 1e) [12,13,14]. At the same time, the mass transfer surface area increased in the same direction [15].

The development of separation, concentration and purification through liquid membranes did not exclude, over time, any of the presented operating variants [16], as each has advantages and disadvantages constantly studied, with technical solutions in continuous development [17].

If we focus on BLM, the technical problems to be solved are: the large volume of solvent used (V), the small mass transfer area (σ), the almost unit ratio between the volume of the source phase, the volume of the receiving phase (r) and the volume of the membrane organic solvent (OS or M) and therefore, implicitly, the long operating time (t) [18,19,20].

Bulk liquid membranes (BLM) are constituted as a stage of laboratory study of the transfer and/or separation of some chemical species of interest. BLMs allow, in a relatively short time, the determination of the individual competitive influence of the operating parameters: pH, concentration of chemical species, ionic strength, concentration of some complexes or carriers, working temperature and the nature of the membrane solvent [21,22,23,24] but also of the configuration or design of the permeation module (geometric shape of the permeator, how to ensure the convection and contact the aqueous phases with the membrane) [25,26].

**Figure 2:**
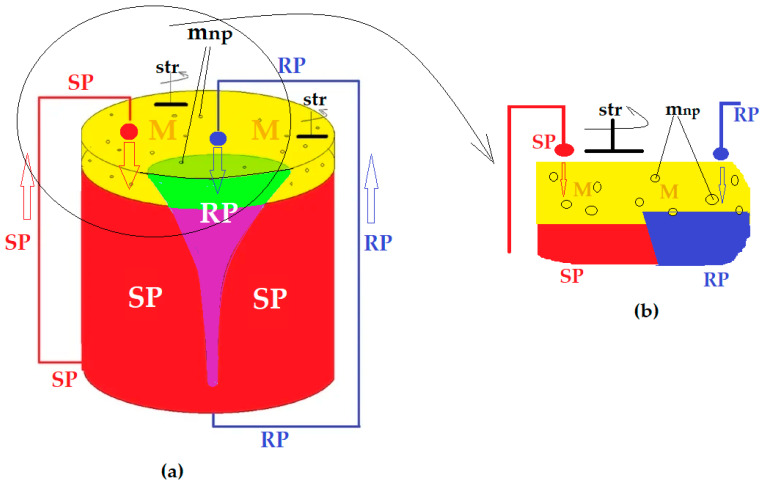
Schematic presentation of the permeation module with dispersed phases: (**a**) general view; (**b**) cross-section detail. SP, source phase; RP, receiving phase; M, organic solvent membrane; m_np_, magnetic nanoparticles; str, stirrer with magnetic rods.

Recently, a BLM system with dispersed phases has been studied, in which the aqueous phases of the separation system disperse in/through the membranes. The membrane is a nanodispersed system of magnetic nanoparticles that has the role of ensuring both convection and transport for ionic chemical species in membranes based on saturated alcohols C_6_–C_12_ [27,28,29,30]. The most recent design is shown in Figure 2 [29,30]. Figure 2a presents the whole permeation module, while Figure 2b details the cross-sectional detail of the permeator. The contribution of this design is the recirculation of aqueous phases as droplets, whose dimensions can be adjusted by the flow rate of the aqueous phases, and the membrane is a dispersion of magnetic nanoparticles set in motion by a rotating magnetic rod (str). This way, the system has all the phases in directed convection [29].

The BLM system with dispersed phases is close to the performances of liquid membranes on hollow fiber support or emulsion-type liquid membranes, but has several limitations that restrict its applicability: stability of the membrane nanodispersion, control of the size of droplets in recirculating aqueous phases, losses of membrane material (solvent or nanoparticles) [30].

In the present paper, a BLM system is approached, starting from the design of the one presented above, in which the recirculation of aqueous phases is performed by flowing on spheres in free rotation. To test the new design of the permeation module, three phenolic derivatives of technical interest and environmental protection were chosen: 4–nitrophenol (NP), 2,4–dichlorophenol (DCP) and 2,4–dinitrophenol (DNP). All of these allow the evaluation of pH as an operational parameter for transport and separation through a membrane consisting of *n*–octanol or *n*–decanol (*n*–AlcM).

## 2. Materials and Methods

### 2.1. Reagents and Materials

All reagents and organic compounds used in the presented work were of analytical grade. They were purchased from Merck (Merck KGaA, Darmstadt, Germany): hydrochloric acid, sodium chloride, sodium hydroxide, *n*–octanol, *n*–decanol, 4–nitrophenol (or *p*–nitrophenol), 2,4–dichlorophenol and 2,4–dinitrophenol (Table 1).

The purified water characterized by 18.2 μS/cm conductivity was obtained with an RO Millipore system (MilliQ^®^ Direct 8 RO Water Purification System, Merck, Darmstadt, Germany).

### 2.2. Methods

#### 2.2.1. Design of the Experimental Installation

**Figure 3:**
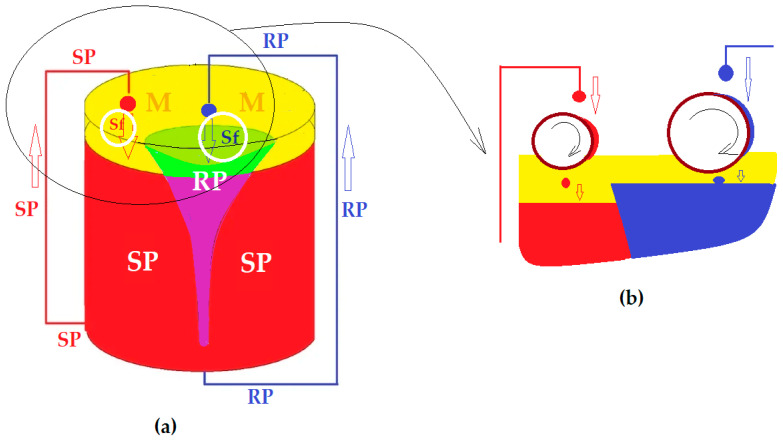
Schematic presentation of the permeation module with dispersed phases with spheres: (**a**) the idea of positioning the spheres; (**b**) how to extend the surface of the aqueous phases from the drop, as a film on the considered spheres. SP, source phase; RP, receiving phase; M, organic solvent membrane; Sf, spheres.

**Figure 4:**
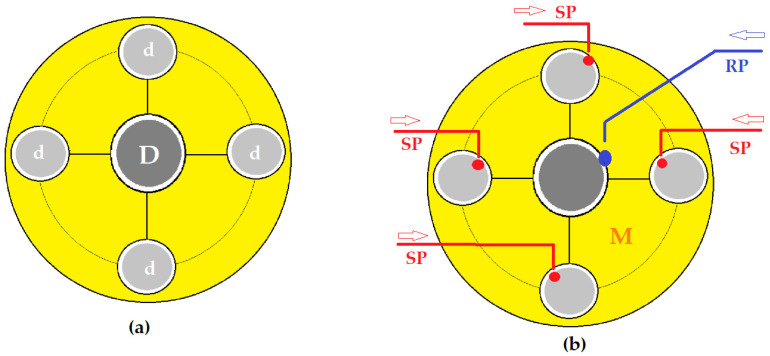
Schematic presentation of the positioning of the distribution spheres (**a**); and of the aqueous phases (**b**). d, spheres for the distribution of the source phase (SP); D, sphere for the distribution of the receiving phase (RP) in the liquid membrane (M).

The permeation module has been previously presented and studied [29,30] and has, as a main characteristic, the recirculation, in the form of drops, of both the aqueous source (SP) and receiving phase (RP). The recirculation is achieved with the help of peristaltic pumps with one channel (for RP) or four channels (for SP) (see Figure 3 and Figure 4).

In order to increase the contact surface between the aqueous phases in the form of droplets, through a membrane made up of pure *n*–octanol or *n*–decanol (*n*–AlcM), in the present experiments, spheres in free rotation were interposed in the direction of the drops towards the membrane (see Figure 3a and Figure 4a), inside a positioning and guiding ring. The spheres float on the membrane of the organic solvent, and when a drop falls on their surface (Figure 3b and Figure 4b), they rotate, dressing in a film of the aqueous phase. The free spheres in rotation, determined by the fall of the drops, are guided by the positioning rings so that they do not move transversely through the membrane and do not sink to the aqueous phases. This way, the spheres ensure the formation of the aqueous phase film—thus increasing the contact surface with the membrane—and convection by shaking the membrane.

The experimental aspect pursued by this design is the increase of the contact surface of the aqueous phases, but also the avoidance of the dispersion of the droplets in the membrane organic solvent. Unlike the experiments presented above [30], in this case, the flow is no longer a limiting parameter. However, in order to compare the performance of the free-falling droplet module, presented above, with that of droplets that are distributed on free-rotating spheres, for the transport of p-nitrophenol, the flow rate was kept constant at 20 mL/min, for only one drip mouth [30].

The four source phase distribution spheres had a diameter (*d*) of 30 mm, and the receiving phase distribution sphere had a diameter (*D*) of 50 mm.

Because the adhesion to the surface of the spheres is an important variable, glass spheres, titanium steel and copper spheres were used for dispersion in the form of film, detachment of the accumulated liquid and its fall from the membrane in the corresponding aqueous phase.

#### 2.2.2. Transport and Separation Experiments of the Phenol Derivatives

For the study of the transport and separation of the chosen phenolic derivatives (Table 1), 20 L stock solution was prepared, with a concentration of 10^−2^ mol/L pure phenolic compound in pure water.

Processing for each experiment at the required pH was performed using concentrated hydrochloric acid (for acidic pH) and sodium hydroxide (for basic pH).

The ionic strength of the source phase was adjusted by the addition of sodium chloride in a gravimetric concentration of 1–5%.

The flows of the phenol derivatives from the source phase were determined at specific time intervals, using Equation (1) [31,33]:

(1) $$J=\frac{M}{S\cdot \Delta t}\;(\mathrm{mg}/(m^{2}\;s))\;\mathrm{or}\;\mathrm{mol}/(m^{2}\;s))$$

with *M* being the permeate mass (g or mol), *S* being the effective surface of the membrane (m^2^) and Δ*t* the time interval (s).

The extraction efficiency (*EE* %) for the phenol derivatives was calculated as follows [33,34], based on the phenolic solution concentration:

(2) $$EE\left(\%\right)=\frac{\left(c_{0}-c_{f}\right)}{c_{0}}\cdot 100$$

with *c_f_* being the final concentration of the solute (phenol derivatives) and *c*_0_ the initial concentration of the solute (phenol derivatives).

The same extraction efficiency could also be obtained based directly upon the absorbance of the considered solutions (phenol derivatives) [34,35], as in Equation (3):

(3) $$EE\left(\%\right)=\frac{\left(A_{0}-A_{s}\right)}{A_{0}}\cdot 100$$

with *A*_0_ being the initial absorbance of the sample phenolic solution and *A_s_* the current absorbance of the sample.

### 2.3. Equipment

The analysis of the content of the studied phenolic compounds was performed at the wavelength characteristic of each form of phenolic derivatives (phenol or phenolate) (Table 1) using CamSpec M550 spectrometer (Spectronic CamSpec Ltd., Leeds, UK). The UV–Vis spectra of the samples were recorded for a wavelength ranging from 200 to 800 nm, at room temperature, using 10 mm quartz cells.

Furthermore, the UV–Vis validation analysis of the phenol derivative solutions was performed on a dual-beam UV equipment–Varian Cary 50 (Agilent Technologies Inc., Santa Clara, CA, USA) at a resolution of 1 nm, spectral bandwidth of 1.5 nm and a scan rate of 300 nm/s. The UV–Vis spectra of the samples were recorded for a wavelength ranging from 200 to 800 nm, at room temperature, using 10 mm quartz cells.

All determinations were performed on the same day, for each scheduled experiment, by two experienced analysts from different laboratories, based on 3 specimens taken for each sample, and to ensure the quality of chemical measurements, the specific EURACHEM guide was followed. [36].

The phenolic derivative content of the membrane was determined on the basis of the mass balance of the three phases of the membrane system [37].

Determination and monitoring of pH were achieved using a conductance cell or combined selective electrode (HI 4107, Hanna Instruments Ltd., Leighton Buzzard, UK) and a multi-parameter system (HI 5522, Hanna Instruments Ltd., Leighton Buzzard, UK) [38].

The surface characteristics of the spheres were determined with a scanning electron microscope (SEM) equipped with a probe for energy dispersive spectroscopy analysis (EDX). Hitachi S4500 system (Hitachi High-Technologies Europe GmbH, Krefeld, Germany) was used [39].

Contact angle measurements for the considered sphere materials (with distilled water or phenol derivative solution) [40] were carried out with a horizontal microscope with video camera (Viola–Shimadzu, Bucharest, Romania).

## 3. Results and Discussion

### 3.1. Brief Argument for Choosing the pH of Aqueous Phases for the Membrane Transport of Weakly Acidic Phenolic Chemical Species

The molecules of the organic substances were transported through an organic liquid membrane by means of a series of solubilization–diffusion equilibria that take place at the two interfaces of the membrane. In the case studied in this paper, the phenolic derivatives considered were organic compounds with a weak acidic character (Table 1). In the acidic source aqueous solutions prepared for experiments, phenolic derivatives were found in a molecular form favorable to the passage of the alcohol membrane in order to concentrate in the alkaline aqueous receiving phase [41].

Phenolic derivatives were distributed between the source phase and the membrane according to the equilibrium in Equation (4):

(4) $${\left(\mathrm{ArOH}\right)}_{F.S.}\;⇌\;{\left(\mathrm{ArOH}\right)}_{M}$$

characterized by the distribution constant, R:

(5) $$R_{\mathrm{ArOH}}=\frac{{\left[\mathrm{ArOH}\right]}_{M}}{{\left[\mathrm{ArOH}\right]}_{F.S}}\cdot 100$$

the process of distribution of phenolic derivatives in the membrane system is influenced by partition-diffusion equilibria, but in aqueous solutions, there are also competitive chemical equilibria involving proton transfer (see Equations (6) and (7)) [42]:

(ArOH)_SP_ + HOH ⇌ (ArO^−-^)_SP_ + H_3_O^+^ (6)

(ArOH)_M_ + (HO-)_RP_ ⇌ (ArO^−^)_RP_ + (HOH)_RP_ (7)

These equilibria could be used, by adjusting the pH of the aqueous phases, for the separation of the three phenolic derivatives considered [43]. Thus, the use of the degree of formation of the chemical species (phenol and phenolate) is required in the case of the considered membrane system: acid source phase–pure alcohol membrane–basic receiving phase.

The degree of formation of one of the chemical species can be defined by a general relation of the form [44]:

(8) $$\alpha_{c}=\frac{\left[H_{c}A^{\left(n-c\right)-}\right]}{C_{H_{n}A}}$$

α_c_ being the degree of formation of species *H_c_A^(n−c)−^*, and *C_HnA_* the total concentration of the phenolic derivative in aqueous solution.

For the phenolic acid derivative, ArOH, of total concentration *C*, as a result of equilibrium (9) in solution, we have the chemical species ArOH (phenol) and ArO^–^ (phenolate) [45]:

ArOH + HOH ⇌ H_3_O^+^ + ArO^−^ (9)

characterized by the acidity constant (10):

(10) $$K_{a}=\frac{\left[H_{3}O^{+}\right]\left[{\mathrm{ArO}}^{-}\right]}{\left[\mathrm{ArOH}\right]}$$

The degree of formation of these chemical species is [46]:

(11) $$\alpha_{1}=\frac{\left[\mathrm{ArOH}\right]}{C}$$

(12) $$\alpha_{0}=\frac{\left[{\mathrm{ArO}}^{-}\right]}{C}$$

Using the mass balance equation of the chemical species present in solution [47], for the phenolic derivative ArOH (acid), the expression of the degrees of dissociation, $\alpha_{1}$ and $\alpha_{0}$ can be easily deduced (Equation (11)):

(13) $$\alpha_{1}=\frac{\left[\mathrm{ArOH}\right]}{C}=\frac{1}{1+10^{pH-pK_{a}}}\;\mathrm{and}\;\alpha_{0}=\frac{\left[{\mathrm{ArO}}^{-}\right]}{C}=\frac{1}{1+10^{pK_{a}-pH}}$$

Theoretical considerations and previous experimental results [48,49,50] have determined the use of a pH in the range 2–6 for the source phase and in the range 10–13 for the receiving phase. Thus, all three phenolic derivatives can be brought in neutral form, which is more soluble in the membrane organic solvent than in the source phase, and in phenolate form, which is more soluble in the aqueous receiving phase than in the membrane.

### 3.2. Transport of p-Nitrophenol in the Free-Rotating Spherical Membrane System

The transport experiments were performed in the installation presented in Figure 2, Figure 3 and Figure 4, both in the known variant [29,30] with the dispersion of the aqueous phases as droplets through the membrane (Md) and by the interposition of the spheres in the direction of the drops through the membrane (Ms). In the second case, the aqueous phases in the form of a film formed on the spheres in free rotation were contacted with the membrane. The volumes of the phases were determined from previous experiments: 5000 mL in the source phase, 500 mL in the receiving phase and 500 mL for the membrane based on *n*–pure alkyl alcohols (*n*–AlcM) [30].

#### 3.2.1. The Influence of the Source Phase pH and of the Nature of the Membrane

**Figure 5:**
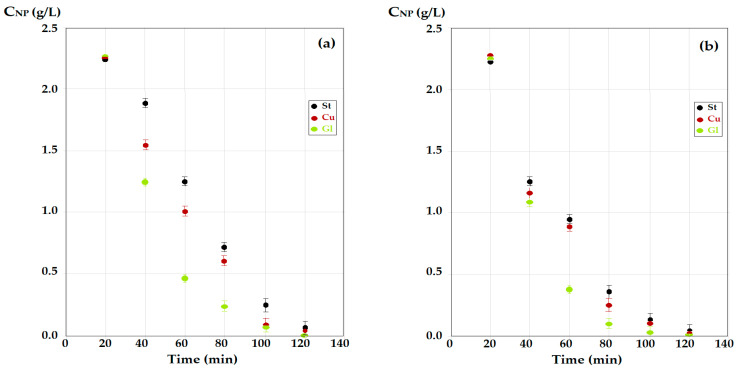
The decrease in concentration of *p*–nitrophenol for operation with the three types of materials: St, steel; Cu, copper; and Gl, glass; (**a**) for source phase with pH = 5; (**b**) with pH = 2; at the same value of the pH of receiving phase (pH = 13), for *n*–octanol membrane.

Under the mentioned experimental conditions, the receiving phase—the sodium hydroxide solution, had a pH of 13, and the source phases of *p*–nitrophenol (approximately 2 g/L) had a pH of 2 or 5. The flow rate of the source phase was 80 mL/min (20 mL/min for each nozzle), and the flow rate of the receiving phase was 20 mL/min. The results obtained (see Figure 5) for the distribution spheres of steel (St), copper (Cu) or glass (Gl) using *n*–octanol as the membrane phase indicated a rapid decrease in the concentration of *p*–nitrophenol in the case of a source phase with pH = 5 (Figure 5a), which was accentuated when the pH dropped to 2 (Figure 5b).

**Figure 6:**
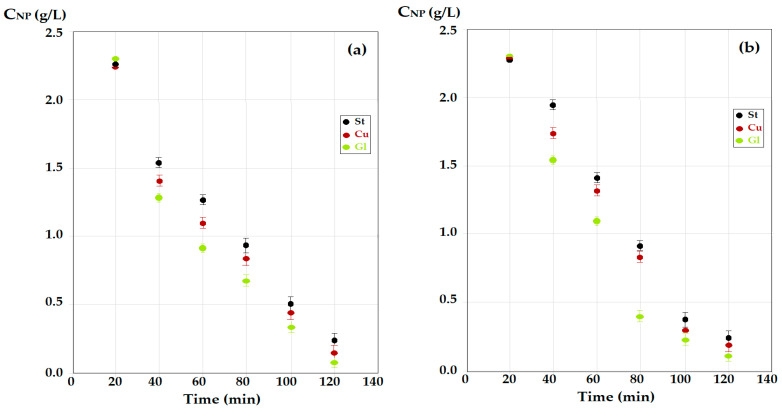
The decrease in concentration of *p*–nitrophenol for operation with the three types of materials: St, steel; Cu, copper; and Gl, glass; for the source phase with pH = 5 (**a**); and pH = 2 (**b**); at the same value of (pH = 13) of the receiving phase, for the *n*–decanol membrane.

Using *n*–decanol as the membrane solvent (Figure 6), the decrease in the concentration of *p*–nitrophenol from the source phase with pH = 5 (Figure 6a) and with pH = 2 (Figure 6b), was slower than in the case of *n*–octanol, for equal operating times (approximately 120 min). Thus, in these experiments (see Figure 6), more phenolic compounds remained in the source phase.

At some point, the results depend on both the nature of the membrane alcohol and the pH of the source phase. The best results of the transport of *p*–nitrophenol were obtained when using a source phase of pH = 2 and a receiving phase of pH = 13, for the case of glass contact spheres, when the membrane consists of *n*–octanol (Figure 5a—the green dots).

#### 3.2.2. Influence of the Nature of the Material for Making Contact Spheres

**Figure 7:**
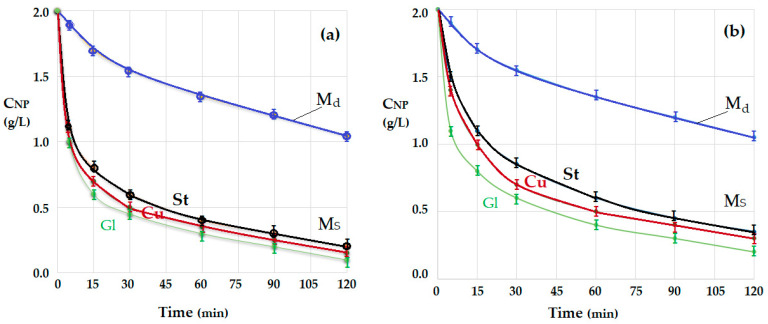
The decrease in concentration of *p*–nitrophenol for operation in the drip module (Md) and in the module with spheres (Ms) of the three types of materials: St, steel; Cu, copper; and Gl, glass; for source phase (SP) with pH = 2 and receiving phase (RP) with pH = 13. (**a**) *n*–octanol membrane; (**b**) *n*–decanol membrane.

Under the established operating conditions (pH = 2 for the source phase, pH = 13 for the receiving phase) for a 2 g/L *p*–nitrophenol solution, transport experiments were performed with the dispersion of the aqueous phases as droplets through the membrane (Md), as well as by interposing the spheres in the path of the droplets through the membrane (Ms) (Figure 7).

The decrease in the concentration of *p*–nitrophenol in the source phase for the *n*–octanol membrane (Figure 7a) was more pronounced than for the *n*–decanol membrane (Figure 7b), both in transport with the dispersion of aqueous phases as droplets through the membrane (Md) and by interposing the spheres in the path of the droplets through the membrane (Ms).

**Figure 8:**
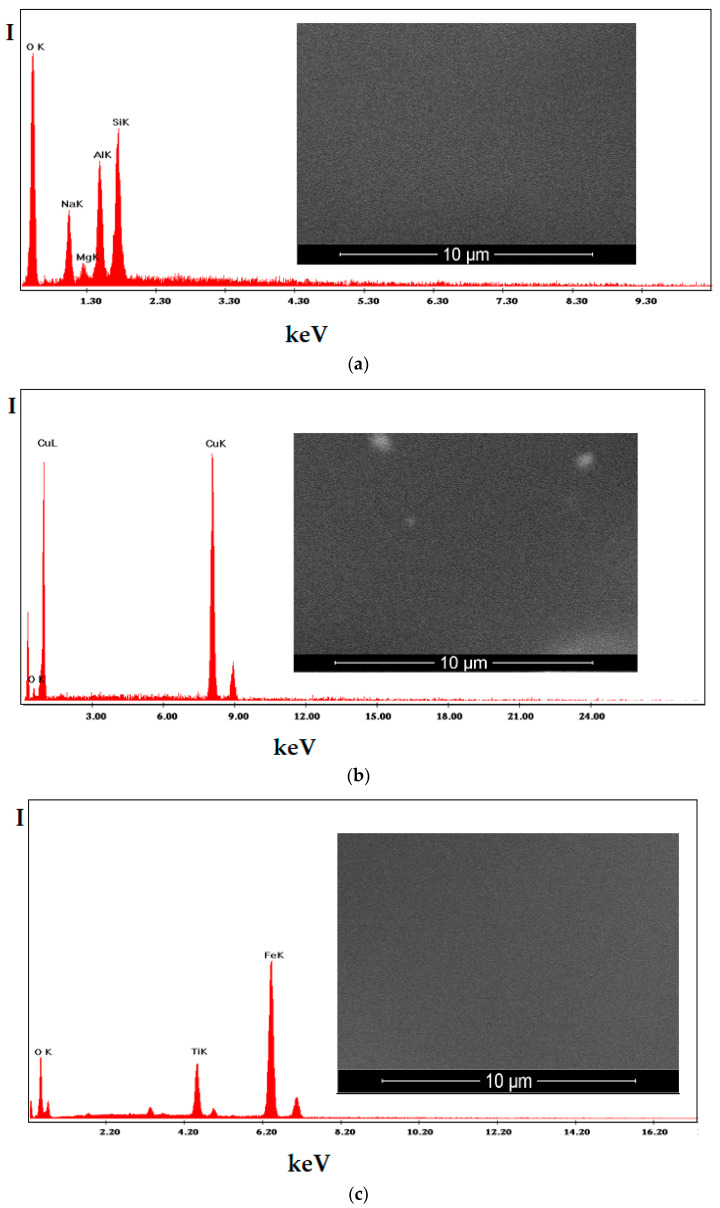
Morphology and composition of the surfaces of the spheres from: (**a**) glass; (**b**) copper; (**c**) titanium steel; obtained by scanning electron microscopy (SEM) and energy dispersive spectroscopy analysis (EDX).

At an operating time of 120 min, the transport results were better for operation in the module using contact spheres (Ms) than for the module using droplet dispersion (Md), for *n*–decanol (Figure 7b), but especially for *n*–octanol (Figure 7a). It is particularly interesting that the glass spheres (Gl) ensured a better contact than the copper ones (Cu), with both being superior to the steel ones (St) (Figure 7). This observation required the analysis of the surface of the contact spheres by scanning electron microscopy (SEM) and energy dispersive spectroscopy analysis (EDX) (Figure 8).

Because the morphological appearance of the surfaces was slightly different (SEM), and the elemental analysis (EDX) showed that on each surface, in addition to the specific elements, oxygen was identified (Figure 8a–c), it was necessary to determine the contact angle of pure water with these oxide surfaces (Table 2).

The contact angles of pure water with the sphere material showed that all three types were hydrophilic, with a contact angle (θ) of less than 90°. The most hydrophilic sphere was the glass one, with the smallest contact angle and the highest oxygen content (oxides). At the opposite pole was the steel, which had the largest contact angle.

The influence of the nature of the material of the contact spheres, therefore of the contact angle, on the decrease of the concentration of *p*–nitrophenol was more visible in the case of the *n*–decanol membrane (Figure 7b).

The use of glass spheres is recommended by the possibility offered by this material to modify the surface with various organic compounds, which can influence the selectivity of the process [51,52]. However, the materials for making the spheres of such a module must remain technically and economically accessible and not disturb the study of the influence of a certain physical-chemical parameter of the proposed membrane system—given that the destination of a module with the design based on bulk liquid membranes remains the laboratory experiment.

The superior transport results of the module using spheres over the droplet module were most likely related to the amplification of the contact surface between the aqueous phases and the membrane phase.

Thus, if we refer to the surface offered by the drip module, for a drop of diameter (*d_d_*) and surface (*S_d_*), we can write Equation (14):

(14) $$S_{d}=\pi d_{d}{}^{2}$$

where the diameter of the droplet is known, (*d_d_*) = 4 ± 2 mm [30].

The number of drops can be obtained knowing the volume of the aqueous phase and the flow rate, but it can also be determined by computerized video means [30,53].

At the same time, it is possible to calculate the surface offered by the transformation of the volume of a drop (15), into a surface film of imposed thickness, on the spheres considered with diameters *d* and *D*, respectively, Equations (16) and (17):

(15) $$V_{d}=\pi d_{d}{}^{3}/6$$

(16) $$S_{fSP}=\pi d^{2}$$

(17) $$S_{fRP}=\pi D^{2}$$

**Figure 9:**
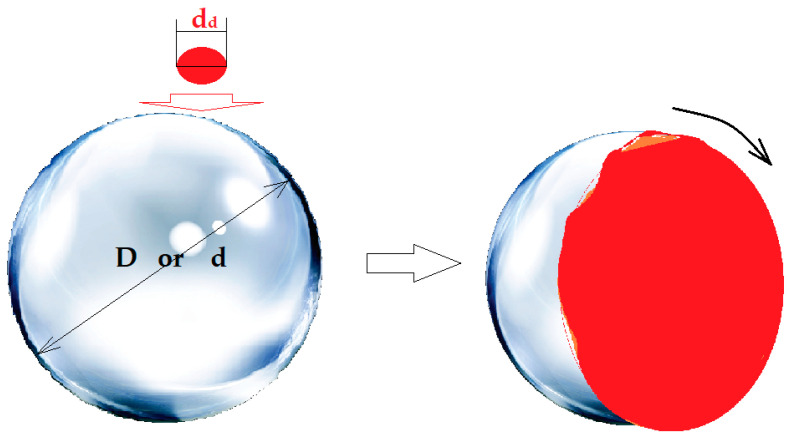
Transformation of the aqueous phase drop into a film on the surface of the considered contact sphere.

Figure 9 illustrates the transformation of the aqueous phase drop into a film on the surface of the considered sphere, simultaneously with the generation of the clockwise rotation of the sphere. Depending on where the drop falls, the direction of rotation can change, as desired.

However, the calculation of the effective mass transfer area, according to Equations (1) and (14)–(17), is affected by the hypothesis of a film of the aqueous phase, of imposed thickness and a degree of coverage of the sphere is difficult to determine experimentally.

The apparent flows of the aromatic derivatives considered in the studied membrane system are presented in Table 3 in order to be compared with data known in the literature [31,44,45,46,47,48,49,50]. The mass transfer surface was approximated with the Equations (14)–(17) and the flow (denoted by J) using Equation (1).

Comparative data show that drop-by-drop and free-rotating sphere systems have flows 10 to 100 times higher than previously obtained data for bulk or supported liquid membranes [49]. Because the transfer area is estimated, clear conclusions can be drawn from further research. This observation is all the more important as data from the literature are obtained for lower feed concentrations [31,49].

#### 3.2.3. Influence of Sodium Chloride Concentration on *p*–Nitrophenol Extraction Efficiency

The reduction of the membrane solvent losses but also the decrease of the solubility of *p*–nitrophenol in the source phase can be achieved by adding salts [29]. In order not to introduce a new anion into the system, the salinity is adjusted with 1–5 wt% sodium chloride. Under the specified experimental conditions, after an operating time of 150 min, with a pH = 5 for the source phase and pH = 11 for the receiving phase, the efficiency of *p*–nitrophenol extraction is presented in Table 4.

The choice of less aggressive pH conditions was imposed by the need to reduce the consumption of hydrochloric acid and sodium hydroxide, but also to be able to observe the influence of ionic strength, because at more aggressive pH values (pH = 1 for source phase and pH = 13 for receiving phase) extraction efficiency was nearly quantitative.

The values obtained in Table 4 show that a relatively moderate concentration of sodium chloride (4–5%) in the source phase can compensate for the lower pH difference between the aqueous phases, the optimal working option being at the disposal of the technological operator.

### 3.3. The Transport and Separation of the Phenol Derivatives

2,4–dinitrophenol (DNP) and 1 g/L 2,4–dichlorophenol (DCP) are phenolic derivatives of high toxicity [54], relatively high solubility in water [55] (Table 1) and a very high probability of occurrence as environmental pollutants [56], as they come from the hydrolysis of widely used commercial compounds: their acetylated derivatives [57].

The transport experiments for a duration of 120 min, to evaluate the influence of the pH of the source phase, were performed in the installation according to Figure 2, Figure 3 and Figure 4 in the variant with the interposition of glass spheres in the direction of the drops towards the membrane (Ms). The concentration of the source phase was maintained at 2 g/L phenolic derivatives, respectively, 1 g/L 2,4–dinitrophenol (DNP) and 1 g/L 2,4–dichlorophenol (DCP).

The volume of the phases was set at 5000 mL for the source phase, 500 mL for the receiving phase of pH = 13 and 500 mL for the membrane based on *n*–octanol.

In order for the source aqueous solutions to contain 2,4–dinitrophenol (DNP) and 1g/L 2,4–dichlorophenol (DCP) in a molecular (neutral) form favorable to extraction, from Equations (9)–(19), it follows that a strong acid pH must be used.

**Figure 10:**
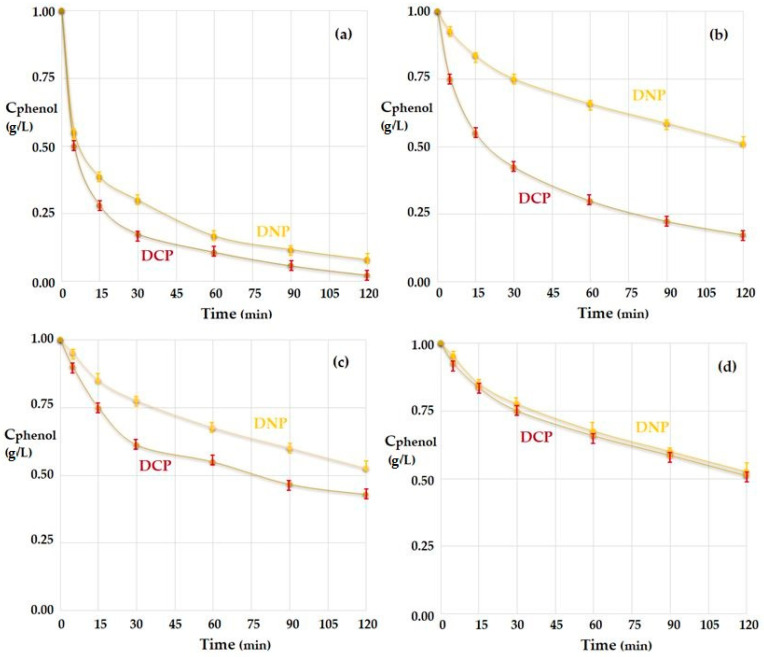
The decrease in concentration of 2,4–dinitrophenol (DNP) and 2,4–dichlorophenol (DCP) for operation with the glass spheres module (Gl), of a source phase with: pH = 2 (**a**); pH = 4 (**b**); pH = 6 (**c**); and pH = 8 (**d**); for a receiving phase with the same pH (pH = 13), for *n*–octanol membrane.

The experimental results (Figure 10) obtained by varying the pH of the source phase from 2 to 8 confirmed that the extraction of the two phenolic compounds could be performed at strong acidic pH (Figure 10a).

Increasing the pH of the source phase to a value of 4, close to pK_a_ 2,4–dinitrophenol (DNP), led to a half-attenuation of the decrease in the concentration of this compound (Figure 10b). Further increasing the pH of the source phase to 6 also affected the reduction of the concentration of the other phenolic compound, 2,4–dichlorophenol (DCP) (Figure 10c).

At a pH = 8 of the source phase close to pK_a_ of 2,4–dichlorophenol (DCP) (Table 1), the reduction of the concentration of the two phenolic derivatives is strongly affected. Thus, more than half of the initial amount of both compounds remains in the source solution (Figure 10d).

Table 5 shows the efficiency of separating 2,4–dinitrophenol (DNP) and 2,4–dichlorophenol (DCP) from a source solution of variable pH with a total concentration of 2 g/L, after an operating time of 150 min, in the installation with glass spheres in free rotation, using a pH = 13 receiving solution.

The efficiency of the extraction of phenolic derivatives in the installation with spheres in free rotation is strongly influenced by the pH of the source phase (see Table 5).

Membrane transport can be amplified by permeation module design parameters, which can improve convection, but being a solubilization–diffusion process, the solubility of phenolic derivatives is very important (Table 1), especially for extraction efficiency.

The solubility of the phenolic derivative, *S_Ar-OH_*, is dependent on pH, according to Equation (18), which shows the available pH range dependencies (before or after pKa of the considered aromatic derivatives):

(18) $$S_{\mathrm{Ar}\text{-}\mathrm{OH}}=\frac{S_{01}}{1+\frac{K_{a,\mathrm{Ar}\text{-}\mathrm{OH}}}{\left[H_{3}O^{+}\right]}}\;\mathrm{or}:\;S_{\mathrm{Ar}\text{-}\mathrm{OH}}=\frac{S_{02}}{1+\frac{\left[H_{3}O^{+}\right]}{\;K_{a,\mathrm{Ar}\text{-}\mathrm{OH}}}}$$

with *K*_*a*,Ar-OH_ being the acidity constant of the corresponding phenolate form.

Thus, the pH dependence is of the general form presented in Equation (19):

*S*_Ar-OH_ = *S*_0,Ar-OH_
*f* (pH) (19)

with *S*_Ar-OH_ being the solubility of the aromatic derivative at a given pH, and *S*_0,Ar-OH_ the solubility in pure water at a considered pH.

**Figure 11:**
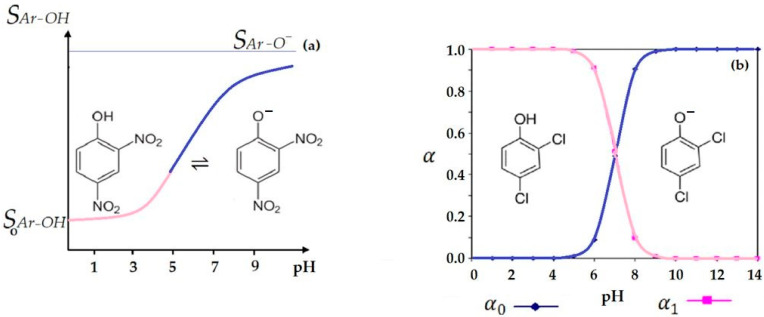
The behavior of phenolic derivatives in the aqueous environment: (**a**) solubility variation of 2,4–dinitrophenol (DNP) depending on pH; (**b**) the degree of formation of 2,4–dichlorophenol (DCP).

Figure 11a shows the evolution of the solubility of the phenolic derivative. Thus, the solubility of the phenolic derivative decreases from pH = 7 (when we have the solubility of S_0_ in pure water) to the limit solubility of the phenolic form at pH = 0 (Figure 11a—red part). On the other hand, from the solubility of S_0_ in pure water (pH = 7), the solubility of the aromatic derivative increased with increasing pH to the limit solubility of the phenolate form at pH = 14 (Figure 11a—blue part). Moreover, the considerations were closely related to the phenol–phenolate balance illustrated by the individual degrees of formation (Figure 11b).

**Figure 12:**
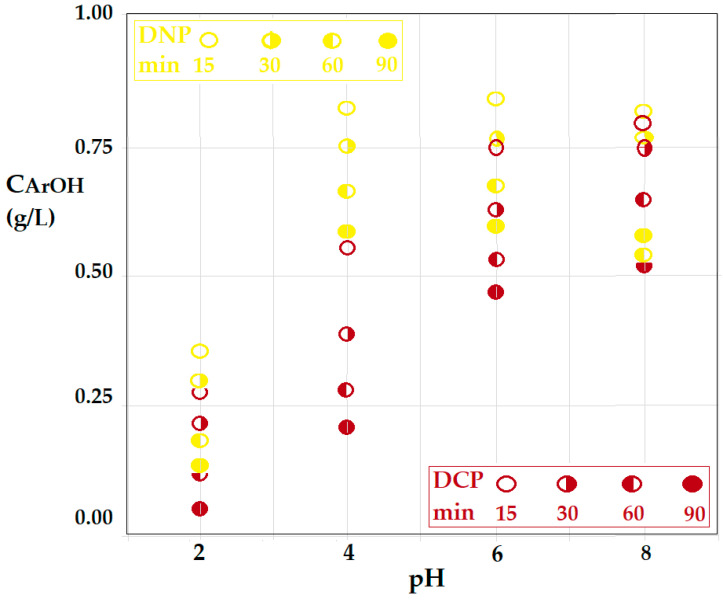
The pH dependence of the source phase concentration for the two aromatic derivatives (2,4–dinitrophenol and 2,4–dichlorophenol), at four representative extraction times: 15; 30; 60 and 90 min.

During the extraction process, the two phenolic derivatives have the behavior illustrated in Figure 12, which shows the effect of pH on the decrease of the concentration of phenolic derivatives at four randomly selected extraction times.

Figure 12 presents the evolution of the source phase concentration for the two aromatic derivatives (2,4–dinitrophenol and 2,4–dichlorophenol) as a function of pH at four representative extraction times: 15, 30, 60 and 90 min. Thus, with increasing pH, the source phase remains with more phenolic derivatives in the composition: 2,4–dichlorophenol (in purple) more than 2,4–dinitrophenol (in yellow). This is because, with increasing pH, phenolate forms become predominant in the source phase, preventing the extraction in the organic membrane phase. Of course, the increase in extraction time contributes to the removal of the aromatic phenolic derivative (the amount of phenol in the source phase decreases).

Starting from the obtained results for the separation and transport of the chemical species in a permeation module with the dispersion of aqueous phases in the form of droplets, this paper studied the effect of interposing spheres in free rotation between the droplets and the membrane phase. These spheres determine the contact, in the form of a film, between the aqueous phases and the membrane phase of the organic solvent. The study was performed in a membrane system in which the variable pH source phase consists of phenolic derivatives (PD) of technical-economic and environmental interest (4–nitrophenol (NP), 2,4–dichlorophenol (DCP) and 2,4–dinitrophenol (DNP)), a membrane of *n*–alkyl alcohols (*n*–octanol and *n*–decanol) and the receiving phase of high pH aqueous solution.

## 4. Conclusions

Starting from the obtained results for the separation and transport of the chemical species in a permeation module with the dispersion of aqueous phases in the form of droplets, in this paper, we studied the effect of interposing spheres in free rotation between the droplets and the membrane phase. These spheres determine the contact of aqueous phases in the form of a film with the membrane phase of the organic solvent. The study was performed in a membrane system in which the variable pH source phase consists of phenolic derivatives of technical-economic and environmental interest (4–nitrophenol (NP), 2,4–dichlorophenol (DCP) and 2,4–dinitrophenol (DNP)), a membrane of *n*–alkyl alcohols (*n*–octanol and *n*–decanol) and the receiving phase of high pH aqueous solution.

The transport of 4-nitrophenol was determined by the difference in pH between the aqueous phases, the nature of the membrane solvent and the nature of the spherical material. Thus, the transport in which the module has glass spheres (Gl) is superior to that using copper spheres (Cu), but especially to those made of steel (St). In all the studied cases, the module with spheres (Ms) had superior transport results to the module with drops (Md).

The extraction efficiency (EE) and the transport of 2,4–dichlorophenol (DCP) and 2,4–dinitrophenol (DNP), studied in the module with glass spheres, showed that the two phenolic derivatives could be separated by adjusting the pH of the source phase. At a source phase with a strong acid character (pH = 2), the two derivatives are extracted with good results (EE > 90%), while at a pH in the range [4,6], they can be separated, with DCP having doubled separation efficiency compared to DNP. At pH = 8 of the source phase, the extraction efficiency halves for both phenolic compounds.

The influence of the material of the spheres in the extraction module suggests possibilities to control the performance of the membrane process by chemically modifying the surface of the spheres, especially those made of glass.

## Figures and Tables

**Table 1:** The characteristics of the used organic compounds [31,32].

Phenol Derivative (Ar-OH)	Name and Symbol	Molar Mass (g/mol)	Absorbance λ (nm)		Solubility in Water (g/L)	pKa
			Ar-OH	Ar-O^–^		
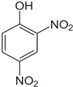	2,4–dinitro phenol (DNP)	185.106	358	361	17.0	4.11
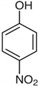	4–nitrophenol (NP)	139.110	317	404	18.1	7.01
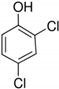	2,4–dichloro phenol (DCP)	161.964	208	284	4.5	7.89
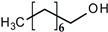	*n*–octanol (nO)	130.230	197		0.300	15.21
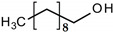	*n*–decanol (nD)	158.280	192		0.037	16.84

**Table 2:** Contact angle for the considered spheres and the atom ratio for key elements.

Sphere Material	Steel (St)	Copper (Cu)	Glass (Gl)
Contact angle (θ°)	83 ± 2	58 ± 2	36 ± 2
Atom ratio (Fe/O, Cu/O, and Si/O)	1:1.12	1:1.47	1:4.22

**Table 3:** Comparative flows of aromatic derivatives through membrane systems.

Liquid Membrane Module	Bulk or Supported [31,40,41,42,43,44]	Drop by Drop	Free Rotating Spheres
Flow (J) (mol/(m^2^ s))	(1–100) 10^−11^	(2.1–5.8) 10^−8^	(5.3–10.6) 10^−8^

**Table 4:** *p*–nitrophenol extraction efficiency (EE) depending on NaCl source phase concentration (after 150 min of extraction).

NaCl (%)	1	2	3	4	5
EE (%)	83 ± 3	86 ± 3	90 ± 3	93 ± 3	95 ± 3

**Table 5:** 2,4–dinitrophenol (DNP) and 2,4-dichlorophenol (DCP) extraction efficiency (EE), depending on the pH of the source phase, after 150 min of extraction.

pH Source Phase		2	4	6	8
EE (%)	DNP	91 ± 3	49 ± 3	48 ± 3	47 ± 3
	DCP	93 ± 3	86 ± 3	61 ± 3	49 ± 3

## Data Availability

Not applicable.
